# Catalyzed Synthesis and Characterization of a Novel Lignin-Based Curing Agent for the Curing of High-Performance Epoxy Resin

**DOI:** 10.3390/polym9070266

**Published:** 2017-07-04

**Authors:** Saeid Nikafshar, Omid Zabihi, Yousef Moradi, Mojtaba Ahmadi, Saba Amiri, Minoo Naebe

**Affiliations:** 1 Department of Applied Chemistry, Faculty of Chemistry, University of Tabriz, Tabriz 51666, Iran; S_nikafshar@yahoo.com; 2 Institute for Frontier Materials, Deakin University, Geelong VIC 3216, Victoria, Australia; mojtaba.ahmadi@ce.iut.ac.ir (M.A.); minoo.naebe@deakin.edu.au (M.N.); 3 Department of Organic Chemistry, Faculty of Chemistry, Isfahan University of Technology, Isfahan 8415683111, Iran; yusefmoradi@yahoo.com; 4 Department of Applied Chemistry, Faculty of Chemistry, Urmia University, Urmia 5756151818, Iran; Sabaamiri6767@gmail.com

**Keywords:** renewable epoxy resin, lignin, curing agent, nanocatalyst

## Abstract

In this study, lignin, an aromatic compound from the forestry industry, was used as a renewable material to synthesize a new aromatic amine curing agent for epoxy resin. Firstly, lignin was separated from black liquor and hydroxyl groups were converted to tosyl groups as leaving groups. Then, primary amination was conducted using an ammonia solution at high pressure and temperature, in the presence of a nano-alumina-based catalyst. The structure of the nanocatalyst was confirmed by FT-IR, ICP, SEM, and XPS analyses. According to the FT-IR spectra, a demethylation reaction, the substitution of hydroxyl groups with tosyl groups, and then an amination reaction were successfully performed on lignin, which was further confirmed by the ^13^C NMR and CHNS analyses. The active hydrogen equivalent of aminated lignin was determined and three samples with 9.9 wt %, 12.9 wt %, and 15.9 wt % of aminated lignin, as curing agents, were prepared for curing the diglycidyl ether of bisphenol A (DGEBA). The thermal characteristics of the curing process of these epoxy samples were determined by DSC and TGA analyses. Moreover, the mechanical performance of the cured epoxy systems, e.g., the tensile strength and Izod impact strength, were measured, showing that in the presence of 12.9 wt % aminated lignin, the mechanical properties of the aminated lignin-epoxy system exhibited the best performance, which was competitive, compared to the epoxy systems cured by commercial aromatic curing agents.

## 1. Introduction

Due to increasing concerns associated with environmental problems such as greenhouse gas emissions, bio-polymers have attracted great attention as a substitution for petroleum-based polymers [1,2]. Natural and renewable resources such as lignin [3], starch [4], poly lactic acid [5], and cellulose [6] are potential candidates for the synthesis of bio-based industrial polymers. Epoxy resins provide a wide range of applications including protective coatings, constructions, components for the electrical and electronic industry, composites, and adhesives due to having a combination of excellent chemical and mechanical properties [7,8]. In the presence of curing agents, epoxy resins form cross-linked networks in which the reactions of the curing process are irreversible. Several kinds of chemical compounds such as aliphatic and aromatic amines, anhydrides, mercaptans, and phenols can be used to cure epoxy resins [9,10,11]. Each curing agent has a specific curing profile including the time and temperature, leading to the formation of epoxy networks possessing various physical and mechanical features. For instance, aliphatic amines can cure epoxy resin at room temperature, while the curing of epoxy resins using anhydrides requires higher temperatures [12]. As petroleum-based aliphatic and aromatic amines are generally hazardous materials, they can potentially cause some health and environmental problems [13,14]. Therefore, it is necessary to synthesize and develop curing agents from renewable materials to replace petroleum-derived materials with bio-based compounds. Although several bio-based compounds that are able to cure epoxy resins have been found, their numbers are limited, and most importantly, their thermo-mechanical properties are not comparable with petroleum-based curing agents. For instance, furanic amines synthesized from polysaccharides and sugars suffer from low reaction yields [15,16,17]. Moreover, most bio-based amines, which can be employed as curing agents are amino acids, which are synthesized by either enzymatic or fermentation methods [18]. Lysine [19,20] and tryptophan [21,22] amino acids as curing agents in curing DGEBA have been reported, with studies suggesting that their glass transition temperatures (*T*_g_) and thermal degradation temperatures are significantly lower than those of petroleum-based curing agents. Furthermore, bio-based anhydrides, as curing agents for epoxy resin, can also be synthesized from various bio-sources such as terpene [23] and rosin [24]. Qin et al. [25] used rosin-based anhydride to cure epoxy resin and the resulting epoxy system had both excellent thermal and mechanical properties. Tannic acid was used as a phenolic curing agent whose results showed that the tensile strength and *T*_g_ of the cured epoxy system using this curing agent were lower than those of commercial systems [26]. Aminated grape seed oil was utilized as a polyamine curing agent which formed a cured epoxy network possessing a very low *T*_g_ (−38 °C), categorized as an amorphous thermoset polymer [27]. Phenalkamine was extracted from cardanol by the Mannich condensation reaction in the presence of formaldehydes and amines, and it was applied as an amine curing agent to cure epoxy resins [28,29]. Although this type of curing agent is set to be deployed as a commercial bio-based curing agent, formaldehyde compounds are hazardous and carcinogenic reactants. Darroman et al. [30] synthesized a new aromatic bio-based amine from cardanol without using formaldehyde, but the thermal and mechanical properties of its cured epoxy network were not satisfactory.

Wang et al. [31] synthesized aminated lignin by the Mannich reaction and it was applied to decolorize anionic azo-dyes. Adding amine groups to lignin increases both lignin’s solubility in water and its reactivity with nucleophiles. Moreover, lignin-based amines synthesized via the Mannich reaction were used in waterborne polyurethanes [32]. It was reported that various kinds of aminated lignin improved the mechanical properties and aging resistance of polyurethane systems. In another study, lignin, after being treated with epichlorhydrin and amination by the Mannich reaction, was used to adsorb heavy metals from aqueous solutions [33]. By grafting alkaline lignin with methyl amine and formaldehyde, a Mannich base biosorbent was prepared [34]. In addition, the Mannich reaction was applied to the amination of lignin for different purposes [35,36,37,38].

**Figure 1:**
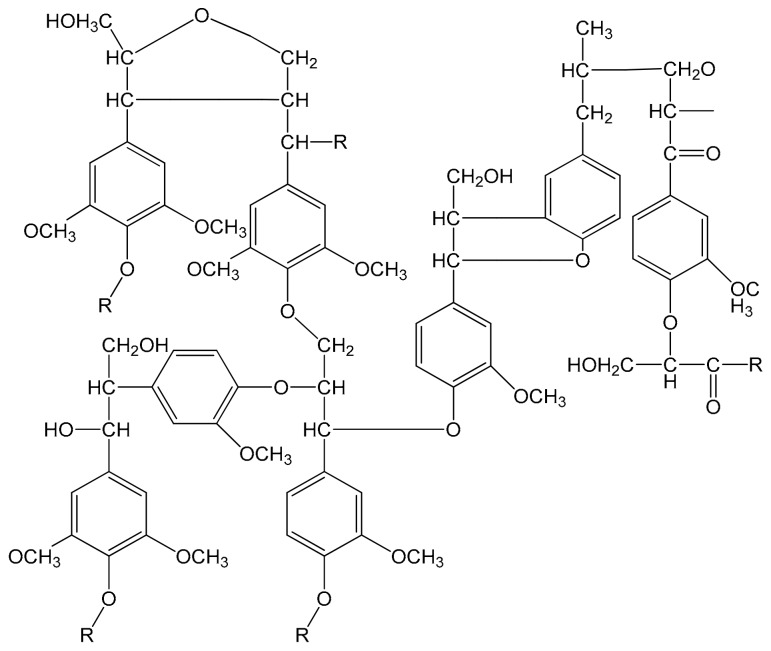
Proposed chemical structure for lignin [48].

Lignin is the second most plentiful renewable polymer in the earth after cellulose and it can be extracted from wood and annual plants using various extraction methods [39]. Lignin is classified as a phenolic polymer and its chemical structure includes phenylpropane units derived from three aromatic alcohol precursors (monolignols) consisting of *p*-coumaryl, coniferyl, and sinapyl alcohols [40,41]. The chemical structure of lignin is shown in Figure 1. In several studies, epoxy resins have been synthesized by the epoxidation of lignin [42,43,44]. In addition, it has been used to synthesize phenolic resins, adhesives, polyolefins, and other miscellaneous applications [45]. Pan et al. [46] made a lignin-based epoxy resin from the reaction of epichlorohydrine with lignin and then epoxied lignin was reacted with propane diamine to create a cured epoxy system. The prepared lignin curing agent in the mentioned study possesses a low number of amine groups, requiring a co-curing agent for the full-curing of epoxy resin. Moreover, the lignin-based epoxy resins usually have a low *T*_g_ and thermal stability because the low number of epoxy rings hinders the formation of a high crosslink density in the cured epoxy system. Therefore, the synthesis of lignin-based curing agents may be preferable to prepare high performance epoxy systems. In this regard, few studies have been done, revealing that the prepared lignin curing agent possesses a low amount of amine groups, requiring a commercial co-curing agent for the full-curing of epoxy resin [47]. In this study, primary amine groups were directly introduced into the lignin structure using a synthesized nanocatalyst of cobalt/copper supported on nanoalumina to enhance the hydrogen amine equivalent of a lignin-based curing agent. Then, this synthesized lignin-based curing agent was used to cure the Diglycidyl Ether of Bisphenol A (DGEBA) epoxy resin, and its thermal and mechanical properties were investigated.

## 2. Experimental

### 2.1. Materials

Liquid Diglycidyl Ether of Bisphenol A (Epon 828), with an epoxy equivalent weight of 185–192 g/eq, was purchased from E.V Roberts (Carson, CA, USA). Black liquor containing 43 wt % waste lignin was supplied from the Kraft pulping of spruce (Choka Company, Anzali, Iran). Sodium sulfite (98%), sodium hydroxide (97%), hydrochloric acid (36.5%), pyridine (99%), *p*-toluenesulfonyl chloride (99%), 1,2 dichloroethane anhydrous (99.8%), and hydrazine hydrate (98%) were purchased from Sigma Aldrich. Cobalt (II) chloride hexahydrate (99%), cobalt (II) nitrate hexahydrate (99%), copper (II) nitrate hexahydrate (99.5%), and ammonia solution (25%) were supplied from Merck, Darmstadt, Germany. Nano gamma alumina (99%, average particle size 20 nm, specific surface area 138 m^2^/g) was purchased from US Research Nanomaterials Inc.

### 2.2. Methods

#### 2.2.1. Synthesis of Nanocatalyst for Amination Reaction

Firstly, 25 g nanoalumina, 2.5 g Co(NO_3_)_2_·6H_2_O, and 2.5 g Cu(NO_3_)_2_·6H_2_O were mixed with 100 mL distilled water and the mixture was then poured into a 1 L reactor (batch reactor, Payafanavar), followed by stirring at 200 rpm for 4 h at 140 °C. After 2 h, 40 g hydrazine hydrate, as a reducing agent, was added to the mixture. Afterwards, the total mixture was filtrated and the obtained sediments were placed in an oven at 100 °C for 1 h, and then calcined in a furnace at 400 °C for 3 h.

#### 2.2.2. Separation Lignin from Black Liquor

In order to separate the lignin from black liquor by ultrafiltration, it was necessary to decrease its viscosity by diluting it with an aqueous solution of acetic acid of 50% (volume ratio: 1:3). To remove the impurities, 100 mL diluted black liquor was poured in a dead-end filtration cell under nitrogen pressure to drive the liquor through the lab-made membrane. The membrane was made from 18% polyethersulphone containing 1% polyvinylpyrrolidone. The remaining lignin was collected and put in the oven at 40 °C for 24 h to obtain a completely dried lignin powder.

#### 2.2.3. Synthesis of Lignin-Based Curing Agent

The synthesis of the lignin-based curing agent consists of three steps, as follows. In the first step, for the demethylation of lignin, 115.1 g lignin, 115.1 g distillated water, and 11.5 g Na_2_SO_3_ were mixed in a 300 mL flask, and 11.5 g NaOH was then added to increase the solubility of lignin. Na_2_SO_3_ acts as a demethylation reagent of lignin. The mixture was stirred at 1000 rpm at 90 °C for 1 h. Then, the mixture was cooled down to room temperature and the pH was adjusted to 2 by a HCl solution of 1% (*v*/*v*). The demethylated lignin was precipitated and separated from the solution by a centrifugation process. Afterwards, it was washed three times with distilled water to reach neutral pH. The demethylated lignin particles were placed in the vacuum oven at 50 °C for 12 h prior to use.

In the second step, since hydroxyl groups are not good leaving groups and are not reactive in substitution reactions, they have to be replaced by strong leaving groups such as OTs. For this purpose, 30 g demethylated lignin was dissolved in 50 mL pyridine, and 100 g 4-toluenesulfonyl chloride was then added to this solution. Afterwards, 13.5 g CoCl_2_·6H_2_O, 100 mL 1,2 dichloroethane anhydrous, and 50 mL distilled water were further added to the total solution and the resulting solution was refluxed at 60 °C for 1 h. Then, the organic phase was extracted and washed two times with a saturated solution of NaHCO_3_, and it was dried in a vacuum oven at 40 °C for 2 h. In the abovementioned reactions, hydroxyl groups were converted to leaving groups of OTs.

**Figure 2:**
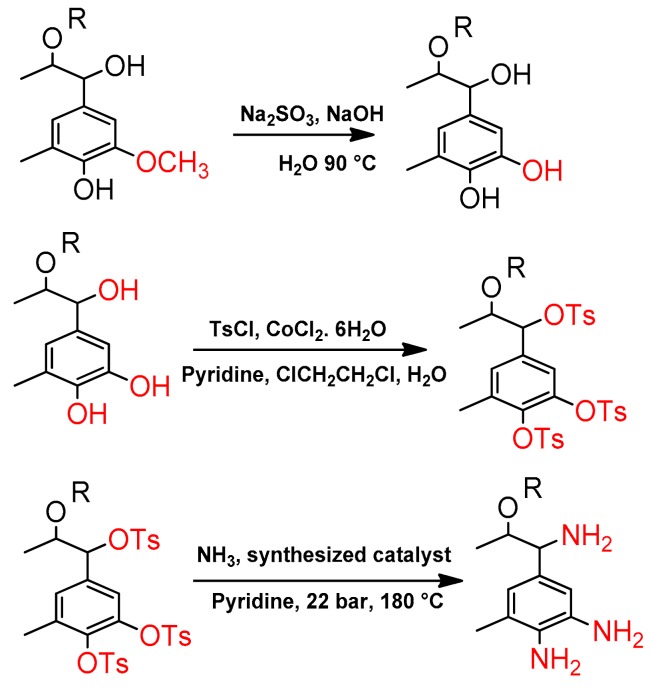
All steps in the synthesis of a lignin-based curing agent.

In the final step, 3.30 g functionalized lignin with OTs obtained from step 2, was dissolved in 50 mL pyridine and 200 mL ammonia solution of 25%, and then 15 g synthesized nanoalumina-based catalyst was added to this solution, before being poured into a high pressure batch reactor. The pressure and temperature were adjusted to 22 bar and 180 °C, respectively, for 2 h under mechanical stirring (IKA RW 20) at 200 rpm. Afterwards, the resulting mixture was cooled down and 50 mL NaOH solution (50 wt %) was slowly added to neutralize the total mixture. To separate the aminated lignin from other side products, a fractionating column was used at 105 °C. In Figure 2, all steps of the synthesis of a lignin-based curing agent are schematically presented.

#### 2.2.4. Epoxy Systems Preparation

Firstly, to utilize the synthesized lignin-based curing agent for the full curing of epoxy resin, its active hydrogen equivalent (AHE) should be calculated. In this regard, the lignin-based curing agent was dissolved in dioxane containing 10% distillated water, and the solution was titrated by a HCl solution of 1 N. Using the following equation, AHE can be determined:

(1) $$\frac{W\times 1000}{A\times N}=\mathrm{AHE}$$

where *A* is mL of consumed HCl, *N* is the normality of the HCl solution, and *W* is the weight of the sample used in titration. The AHE obtained was 24.3 g/eq. Each active hydrogen can react with one epoxy group and the stoichiometric ratio of the synthesized lignin-based curing agent for the reaction with epoxy resin is calculated according to the following equation:

(2) $$\frac{\mathrm{AHE}}{\mathrm{EEW}}$$

where AHE is the active hydrogen equivalent of the curing agent and EEW is the average epoxy equivalent weight of epoxy resin. Therefore, the obtained stoichiometric ratio of the lignin-based curing agent and epoxy resin is 0.129.

To prepare the cured epoxy samples, 100 g epoxy resin was mixed with 12.9 g lignin-based curing agent, dissolved in a small amount of tetrahydrofuran. It is worth mentioning that since lignin is a large molecule, it is possible that some amine groups cannot react with epoxy rings mainly due to the steric hindrance. Therefore, cured epoxy samples with lower and higher stoichiometric ratio values were also prepared as follows: sample A 9.9 wt %; sample B 12.9 wt %; sample C 15.9 wt %. As the melting point of the lignin-based curing agent was too high (>250 °C), it was firstly dissolved in a suitable solvent to react well with the epoxy resin. Various amounts of lignin-based curing agent were dissolved in a small amount of tetrahydrofuran, and then the solution was added into a proper amount of epoxy resin. The mixture was vigorously stirred using a mechanical mixer (IKA RW 20) at 500 rpm for 5 min and degassed under vacuum to remove the solvent for 10 h at 50 °C. The curing reaction was conducted at 125 °C for 2 h, followed by a post curing for 1 h at 150 °C.

### 2.3. Characterizations

The FT-IR analysis was carried out using an FT-IR spectrometer, Tensor 27, (Bruker, Billerica, MA, USA) Bruker with a 40 scan average at a resolution of 4 cm^−1^. The ^13^C NMR spectrum of aminated lignin was obtained at ambient temperature using a Bruker Spectrospin Avanc 400 MHZ NMR. (Bruker Spectrospin, Billerica, MA, USA) Chemical shifts were determined relative to tetramethyl silane (TMS), which was applied as the control. The ^13^C NMR spectrum was acquired with a relaxation delay time of 1 s and a spectral width of 100,000 Hz. The crystalline structure of yjr synthesized nanocatalyst was studied using X-ray diffraction (XRD) with Siemens D5000 (Siemens, Munich, Germany) equipment. To measure and quantify the loaded elements on the gamma nanoalumina as support for the nanocatalyst, inductivity coupled plasma (ICP), using Integra XL equipment, was conducted. Lignin, before and after the amination reaction, was analyzed to determine its elemental composition in the CHNS mode via CHNS Elemental Analyzer Vario EL III machining using helium as a carrier gas, and the amount of oxygen was determined by the difference. The DSC analysis was performed using Linseis, PT10 (Linseis, Robbinsville, NJ) equipment from USA, to investigate the thermal curing characteristics. The DSC machine was firstly calibrated using standard zinc and indium. For each experiment, a 5 mg sample was sealed with an aluminum pan and then tested in the DSC machine using a non-isothermal DSC mode with a heating rate of 10 °C/min under a nitrogen flow of 40 mL/min at a temperature range of −30 to 200 °C. A DSC re-scan was performed on the cured epoxy systems at a heating rate of 10 °C/min to determine the glass transition temperature (*T*_g_). Thermogravimetric analysis (TGA) was performed using TG/DTA 6300 (SII Nanotechnology, Northridge, CA, USA) under nitrogen flow over a temperature range of 0–700 °C at a heating rate of 10 °C/min. A tensile strength test was conducted in accordance with ASTM D638 Type I by using a Shimadzu 20KN-testing (Shimadzu, Kyoto, Japan) machine. Specimen dimensions of samples were selected to be 165 × 19 × 3.2 mm^3^, and the crosshead speed was adjusted to 2 mm/min. At least five specimens were made and tested. The Izod impact strength test was conducted according to an ASTM D256 by a Zwick/Roll 6103 impact tester at room temperature using specimens with dimensions of 63.5 × 12.7 × 7.2 mm^3^. For each sample, five replicates were tested. Fracture surfaces of the cured epoxy systems after the Izod impact strength test and the surface of the synthesized nanocatalyst were observed by employing SEM analyses using a Tescan, MIRA3 FEG-SEM, (Tescan, Kohoutovice) Czech Republic, at an accelerating voltage of 5.00 KV.

## 3. Results and Discussion

### 3.1. Nanocatalyst Characterization

Due to the high surface area, high pore volume, pore-size distribution, acid/base characteristics, local microstructure, and being able to phase combination, γ-nanoalumina is widely used as a catalyst, as well as support for other catalysts, in various industries [49,50]. In the present study, to synthesize the nano size of cobalt and copper metal nanoparticles and apply them as catalysts in the amination reaction, cobalt nitrate hexahydrate and copper nitrate hexahydrate salts were reduced using hydrazine in the porous spaces of γ-nanoalumina. In other words, the synthesized cobalt and copper nanoparticles were trapped in the porous spaces, catalyzing the amination reaction without the dispersion of the reaction in the environment of [51]. It is proposed that the reduction reactions of cobalt nitrate hexahydrate and copper nitrate hexahydrate salts are according to the following reactions:

(3) $$\begin{array}{l} \mathrm{Co}{\left(NO_{3}\right)}_{2}\cdot 6H_{2}O\;+\;3N_{2}H_{4}\overset{{140\;}^{\circ }C}{\to }\mathrm{Co}\;+\;4N_{2}\;+\;12\;H_{2}O\\ \mathrm{Cu}{\left(NO_{3}\right)}_{2}\cdot 6H_{2}O\;+\;3N_{2}H_{4}\overset{{140\;}^{\circ }C}{\to }\mathrm{Cu}\;+\;4N_{2}\;+\;12\;H_{2}O \end{array}$$

**Figure 3:**
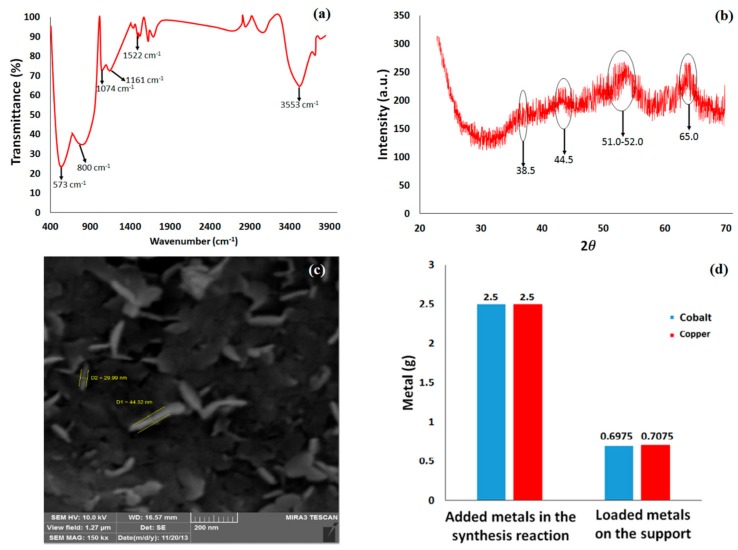
FT-IR spectrum (**a**); XRD pattern (**b**); SEM image (**c**); and ICP analysis (**d**) of the synthesized nanocatalyst.

In order to confirm the chemical structure of the synthesized nanocatalyst, FT-IR spectroscopy was carried out, and its result is shown in Figure 3a. The absorption peaks at 573 cm^−1^ and 800 cm^−1^ are assigned to the stretching mode of AlO_6_, and the absorption peaks at 1074 cm^−1^ and 1161 cm^−1^ are related to the symmetric bending and asymmetric stretching of Al–O–Al, respectively [52,53]. Two absorption peaks at 1522 cm^−1^ and 3553 cm^−1^ can also be assigned to the bending and stretching OH groups, respectively [54]. Vibration peaks around 1000–1200 cm^−1^ are attributed to Cu–O–Al and Co–O–Al, which were overlapped with the asymmetric stretching of Al–O–Al. The XRD pattern of the synthesized nanocatalyst is illustrated in Figure 3b, demonstrating XRD peaks at 2θ of 38.5°, 44.5°, and 65.0°, which are related to gamma alumina nanoparticles [55]. This shows that during the loading of cobalt and copper nanoparticles, the structure of gamma nanoalumina has not been changed. Moreover, the peaks which appeared in the range of 50–52° are assigned to the cobalt and copper nanoparticles [56]. Furthermore, cobalt oxide and copper oxide should only be seen in the XRD pattern if the concentration of these metal oxides is more than 5 wt % with respect to the nanoalumina. Since in the XRD pattern, the peak of these metal oxides cannot be seen, it can be concluded that most of the cobalt and copper ions were completely reduced. However, as mentioned earlier, only 30% of reduced cobalt and copper ions are supported on the nanoalumina surface. Figure 3c shows an SEM image of the synthesized nanocatalyst, illustrating that cobalt and copper nanoparticles are loaded on the nanoalumina surface. The size of the loaded cobalt and copper are calculated to be ~29–44 nm. Inductivity coupled plasma (ICP) analysis provides information regarding the loaded metals on the support. The results of ICP analysis are presented in Figure 3d. As can be seen, the amount of loaded cobalt and copper on the nanoalumina is ~0.7 gr and they are almost equal, which is due to the fact that their reactivity is the same. In addition, only ~30% of the used metal ions were loaded on the nanoalumina.

### 3.2. Lignin-Based Curing Agent Characterization

**Figure 4:**
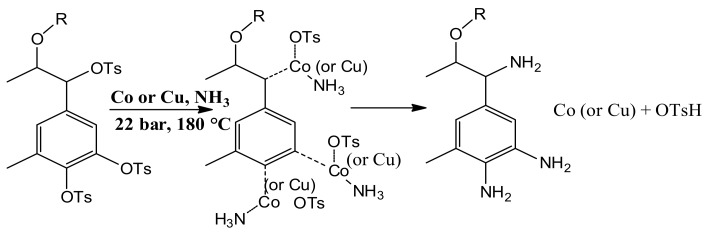
Proposed mechanisms of cobalt/copper catalyzed amination reactions.

Generally, the amination reactions, catalyzed by cobalt/copper-supported on the nanoalumina, include the coordination of the nucleophile to the metal center, activation of the C–X bond (X means leaving group), and C–N bond formation. It is proposed that the used metal nanoparticles act as a catalyst according to the oxidative addition/reductive elimination pathway [57], and its reactions are shown in Figure 4. Herein, the coordination of OT groups with metal nanoparticles provides a better nucleophilic property on their neighbor carbon, which can induce a more nucleophilic attack of ammonium groups. Consequently, ammonium groups can be more easily substituted with OTs, forming a primary amine, whilst cobalt and copper return to the initial transition state and *p*-toluenesulfonic acid is formed. This is in agreement with the literature, which has shown that the reaction of aryl tosylates with ammonia generates amine groups [58,59,60].

**Figure 5:**
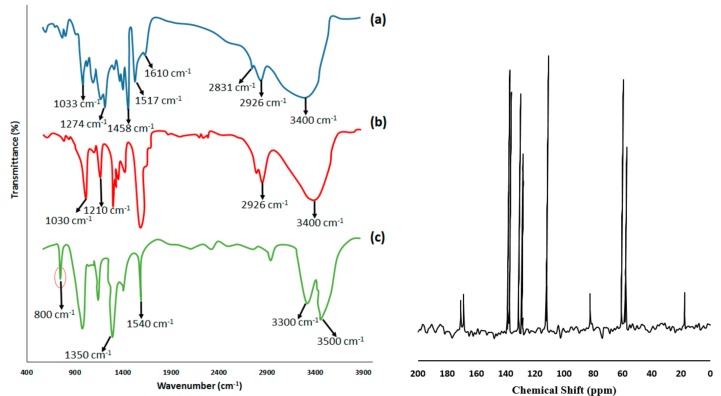
FTIR spectra (**left**) of separated lignin (**a**); demethylated lignin (**b**); and aminated lignin (**c**); ^13^C NMR spectrum (**right**) of aminated lignin.

FTIR spectroscopy was performed to study the chemical structure of lignin before modification, after demethylation, and after amination. Figure 5a presents the FTIR spectrum of the separated lignin from black liquor, before modification. A broad absorption peak around 3400 cm^−1^ corresponds to the stretch vibration of O-H groups. Absorption peaks at 2926 cm^−1^ and 1458 cm^−1^ are assigned to the C–H vibrations of –CH_2_ and –CH_3_ groups, respectively. While, C–H vibration of the –CH_3_O groups shows an absorption bond at 2831 cm^−1^. Moreover, the absorption bonds at 1610 cm^−1^ and 1517 cm^−1^ represent the presence of aromatic C=C vibrations. Absorption peaks that appeared at 1274 cm^−1^ and 1033 cm^−1^ are characteristic peaks of C–O vibrations [61,62]. After the demethylation of lignin, the FTIR spectrum (Figure 5b) shows that the intensity of absorption peaks of -OH and C-O vibrations appearing at 3400 cm^−1^, 1030 cm^−1^, and 1210 cm^−1^ is significantly increased. Moreover, since the number of methyl groups decreased as a result of demethylation reactions, the absorption intensity of the C–H vibration peak at 2926 cm^−1^ reduced. According to this FTIR spectrum, it can be proved that most of the methyl groups are converted to the hydroxyl groups. Figure 5c illustrates the FTIR spectrum of lignin after the amination reaction, presenting two absorption peaks at 3300 cm^−1^ and 3500 cm^−1^ that corresponded to the N–H stretching vibration. Moreover, vibration bonds at 1540 cm^−1^ and 1350 cm^−1^ are related to the N–H bending vibration and C–N vibration, respectively. An absorption peak around 800 cm^−1^ is also assigned to the out of plan N-H vibration [63]. Additionally, there is a medium small peak with a low intensity around 3500 cm^−1^ associated with some remaining hydroxyl groups, which are negligible compared to the introduced amine groups.

The ^13^C NMR of the spectrum of aminated lignin is presented in Figure 5. The peaks around 130 ppm are attributed to the substituted ortho and para carbon sites on the aromatic ring. The peaks at 16.5 and 17.8 ppm are related to methyl groups. The CH group which is connected to the aromatic ring and amine group has a peak at 59.5 ppm. The peak at 82.2 ppm is assigned to CH, which is connected to oxygen. It can be supposed that the peaks at 136.8 and 137.3 ppm are assigned to aromatic carbons which are connected to amine groups. The results of CHNS analysis are also presented in Table 1. The carbon and oxygen contents of pure separated lignin decreased after the amination reaction, while the hydrogen and nitrogen content increased. These changes can be attributed to amination reactions in which hydroxyl and methoxyle groups were converted to primary amine groups. The sulfur content after amination was reduced slightly due to the fact that pure separated lignin was subjected to the purification as a result of dissolving in various solvents.

### 3.3. Thermal Properties of Epoxy Systems

**Figure 6:**
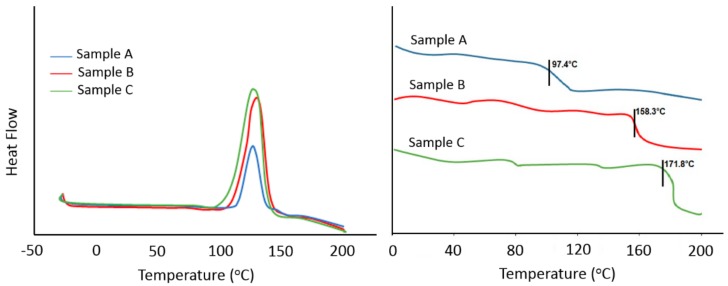
DSC thermograms of various un-cured samples (**left**), and re-scan DSC thermograms of cured samples (**right**) at heating rate of 10 °C/min.

DSC provides useful information about the curing conditions of epoxy systems and many studies have been performed on the epoxy resin to obtain kinetic data using DSC analyses [64,65,66]. The DSC thermograms of three samples are shown in Figure 6 and their results are presented in Table 2. In sample A (9.9 wt % aminated lignin), the onset temperature (*T*_i_) and peak temperature (*T*_p_) are 114.1 and 124.8 °C, respectively, whereas the *T*_i_ and *T*_p_ values of sample B (12.9 wt % aminated lignin) are 102.6 and 129.4 °C, respectively. A comparison on the total heat of the curing reaction (∆*H*) of samples A and B confirms that ∆*H* of sample A is much lower than that of sample B. It is worth mentioning that the opening of epoxy rings by amine groups is an exothermic reaction; therefore, when more epoxy groups are opened in the structure, a higher heat will be released. It means that in sample A, all epoxy groups did not undergo ring-opening reactions, which may be due to the fact that the amount of curing agent in sample A was not sufficient to react with all epoxy groups. The ∆*H* of sample B was about two times higher than that of sample A. Moreover, in sample C, the amount of curing agent increased and consequently the *T*_i_ dropped to 93.9 °C, indicating that the curing reaction of epoxy resin was initiated much sooner than those of samples A and B as a result of more amine groups available to react with epoxy rings at low temperatures. Furthermore, the *T*_p_ of sample C is 121.2 °C, which is lower than that of the peak temperatures of samples A and B, showing that the curing reaction in sample C is completed sooner compared to the other samples. The ∆*H* of sample C is 684.36 J/g, which is higher than that of samples B and A. This means that in sample C, more epoxy groups reacted with amine groups. According to these results, it can be concluded that the amount of curing agent in sample A is not sufficient and the curing reaction of epoxy resin in sample A remains incomplete. On the other hand, no significant differences between samples B and C were observed in terms of their curing characteristics.

The *T*_g_ is directly related to the polymer chain mobility and performance of the curing process, and consequently, the formed epoxy network [67]. As can be seen from Figure 6 (right) and Table 2, the *T*_g_ of sample A (9.9 wt % curing agent) is 97.4 °C, which is much lower than the *T*_g_ of sample B (12.9 wt % curing agent). This tangible difference can be associated to that the fact that the crosslink density in sample A is lower than that of sample B, which may be indicate that sample A is not completely cured. In sample C (15.9 wt % curing agent), *T*_g_ is 171.8 °C, which is 8.5% higher than sample B, while the amount of curing agent used in sample C is 23.2% which is more than the amount of curing agent in sample B. As discussed earlier, lignin is a macromolecule containing a high number of aromatic rings. Therefore, when the amount of aminated lignin used as a curing agent increases, the number of both aromatic rings and amine groups increases, and we would thus expect that the crosslink density of the epoxy structure enhances, and consequently, *T*_g_ is improved. It is worth mentioning that a higher *T*_g_ means a more rigid epoxy network [68,69].

**Figure 7:**
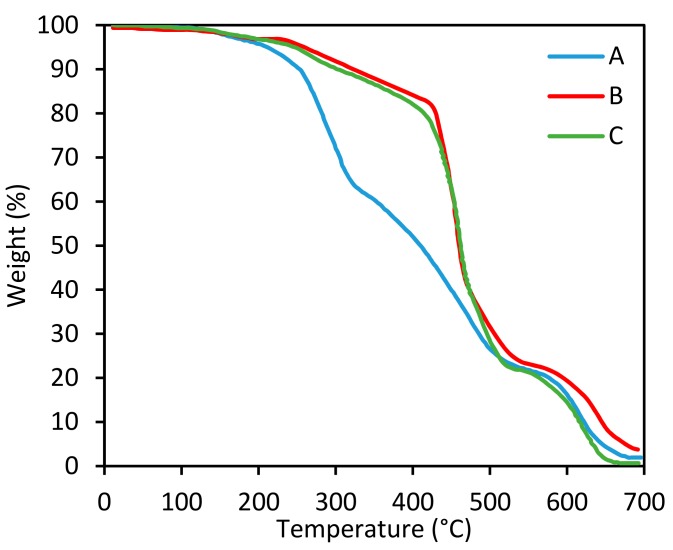
TGA curves of the various samples at a heating rate of 10 °C/min.

The thermal stability of the three samples has also been examined using TGA analysis, and the results are illustrated in Figure 7 and Table 3. As can be seen, there are two degradation stages for all samples, which can be assigned to the degradation of the aliphatic parts (first stage) and aromatic parts (second stage) of the epoxy matrix [70]. Sample A has the lowest onset degradation temperature (*T*_onset_) among the samples, which is due to the low crosslink density and low number of aromatic structures. The *T*_onset_ samples B and C are about 243 and 239 °C, respectively. In sample C, it is supposed that the amount of aminated lignin is excess; therefore, all of them are not entered into the curing reaction. This remaining lignin-based curing agent led to a decrease in the thermal stability of the system.

According to Table 3, the degradation temperature at 5% weight loss (*T*_5%_) of sample A is lower than those of samples B and C. Moreover, sample C has a lower *T*_5%_, compared with sample B. As is expected, the temperature at the maximum rate of degradation (*T*_max_) of sample A is also lower than the *T*_max_ of samples B and C, while samples B and C have similar *T*_max_ values.

### 3.4. Mechanical Performance of Epoxy Systems

According to Table 4, tensile modulus and elongation at the break of sample A are higher than those of other samples. This can be attributed to the more flexible structure in sample A. As it was mentioned, the epoxy matrix in sample A was not fully cured and there are some remaining epoxy groups due to an insufficient amount of curing agent. This leads to a higher elongation at the break for sample A, while the tensile strength of sample A is the lowest among all the samples. On the other hand, sample B has the highest tensile strength, while its elongation at the break is lower than that of sample A. This is due to its higher crosslink density which results in the fact that its chemical bonds cannot be easily elongated under mechanical load compared to sample A. In sample C, both the tensile strength and elongation at the break are lower than those of sample B. It is supposed that due to the higher *T*_g_, the crosslink density in sample C is higher than that of sample A and consequently, it becomes more brittle [65,71]. In addition, it can be argued that the amount of remaining aminated lignin as curing agent in sample C can act as a filler, resulting in an improvement of the tensile strength. Gupta el al. [72] cured DGEBA epoxy resin using 14.5 wt % aromatic amine of metaphenylene diamine and obtained a tensile strength of ~82 MPa. While in the present study, the tensile strength of sample B was 86.1 MPa, which is 5% higher than that of the value reported by Gupta et al. It means that this synthesized lignin-based curing agent can compete with commercially available curing agents in terms of its mechanical properties.

Table 4 also presents the impact of the Izod strength of various samples. In sample A, the Izod impact strength is 27.11 kJ/m^2^, while it reaches 35.73 kJ/m^2^ and 34.29 kJ/m^2^ for samples B and C, respectively. As discussed earlier, this difference can be attributed to the fact that not all of the epoxy groups were cured in sample A; therefore, it can absorb a lower energy compared to the other samples. Here, it can be assumed that sample C is brittle due to the high amount of curing agent; however, an excess amount of curing agent (unreacted aminated lignin) can play a role as a filler and consequently compensates for the brittleness of the epoxy structure to some extent. For this reason, samples C and B have approximately the same Izod impact strength. Fang-Fu et al. [73] cured epoxy resin with 4,4′-diaminodiphenylmethan as the curing agent and reported an Izod impact strength of 6.7 kJ/m^2^, whereas the Izod impact strength of sample A in this study is 433% higher than that of value reported by Fang-Fu et al.

### 3.5. Morphological Observations

**Figure 8:**
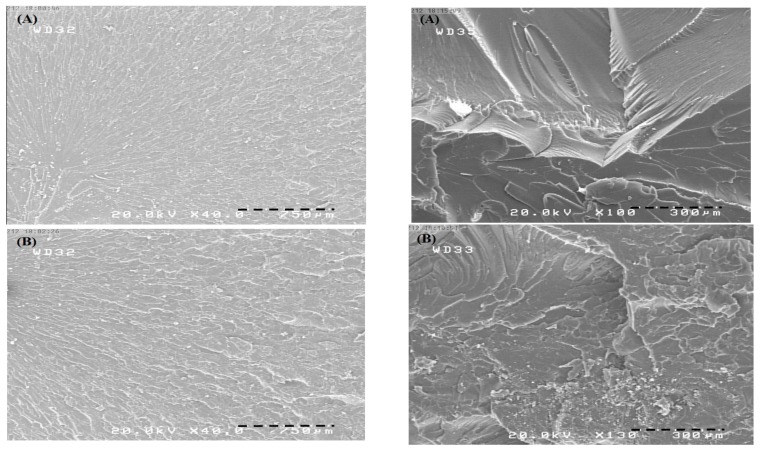

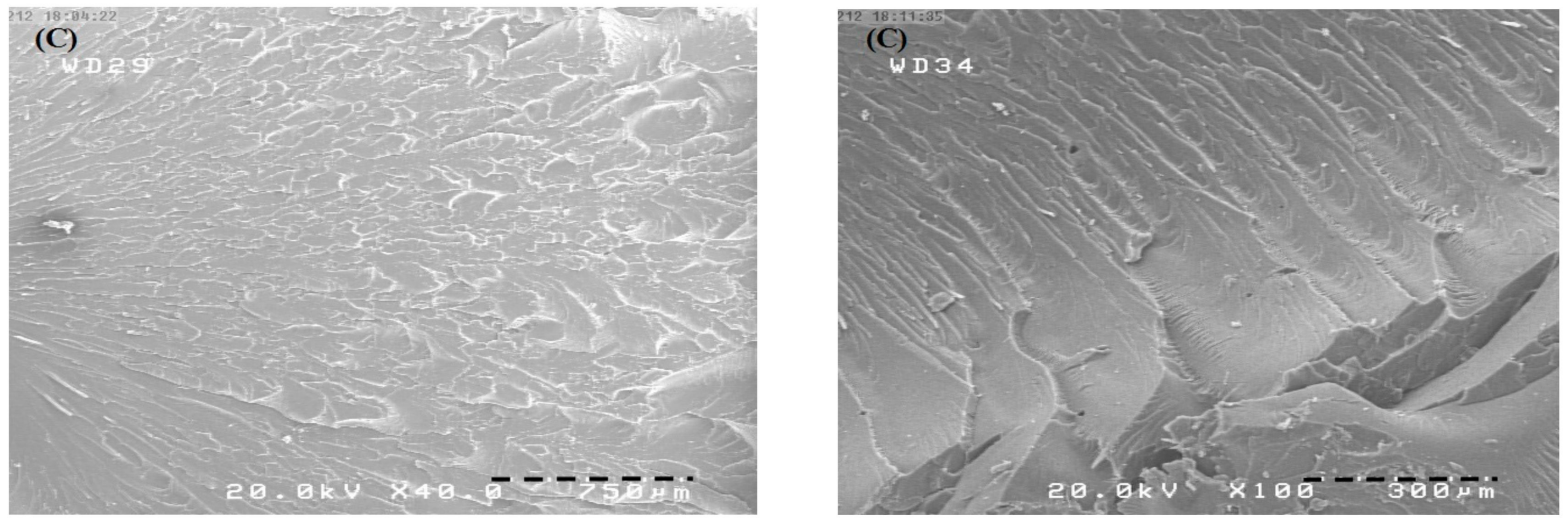
SEM images of the fracture surfaces of the various samples at different magnifications ((**A**) 9.9 wt % curing agent; (**B**) 12.9 wt % curing agent; and (**C**) 15.9 wt % curing agent).

Figure 8 presents the fracture surfaces of various cured samples at two different magnifications (750 µm and 300 µm). As can be seen, the surface of sample A is mostly smooth and only a few fracture surfaces are observed. On the other hand, in sample B, cracks propagate in the labyrinth matrix, causing more fractured plates, and the surface is completely jagged and rough. In sample C, the surface is rough and fractured plates are also present; however, their numbers are lower than that of sample B. It is hypothesized that extra amounts of curing agent were trapped among cured epoxy chains, acting as a filler. Therefore, the surface is rough even though sample C is brittle.

## 4. Conclusions

In summary, the lignin was modified to be used as a bio-based curing agent for epoxy resin. Towards to this end, lignin functional groups were firstly converted to hydroxyl groups through a demethylation process, before being converted to a tosyle-lignin intermediate. Then, an amination reaction was conducted on tosyle-lignin to introduce primary amine groups into the lignin structure, which was catalyzed by a cobalt/copper supported nanoalumina. Using FTIR and ^13^C NMR analyses, the chemical structure of the lignin-based curing agent was successfully confirmed. Moreover, FT-IR spectroscopy was used to identify the chemical structure of the synthesized nanocatalyst. The ICP analysis illustrated that 27.9 wt % and 28.3 wt % of cobalt and copper were loaded on nanoalumina, respectively. The active hydrogen equivalent (AHE) of aminated lignin was then determined by titration to find the stoichiometric ratio of aminated lignin and epoxy resin. The aminated lignin was then used as a bio-based curing agent in the curing of a high performance DGEBA epoxy resin system. The results showed that there is no a direct relationship between the calculated stoichiometric ratio with thermal and mechanical properties. It is argued that since the synthesized lignin-based curing agent is a macromolecule with steric hindrance when involved in the curing process, a higher amount of lignin-based curing agent can change the thermal and mechanical properties. However, according to the obtained results, high *T*_g_ and *T*_onset_ values, as well as a high tensile strength and Izod impact strength, can be confirmed by the application of this synthesized lignin-based curing agent in the preparation of a high performance cured epoxy system, which can compete fairly with the commercially available aromatic curing agents.

## Figures and Tables

**Table 1:** CHNS analyses of lignin before and after amination.

Type of Lignin	Carbon (%)	Hydrogen (%)	Oxygen (%)	Nitrogen (%)	Sulfur (%)
Separated lignin	57.16	4.61	34.17	1.68	1.56
Aminated lignin	55.87	7.58	29.82	4.63	1.39

**Table 2:** Thermal characteristics of the curing process of the various samples obtained from DSC analyses at a heating rate of 10 °C/min.

Sample Code	Curing Agent (Phr)	*T*_i_ (°C)	*T*_p_ (°C)	∆*H* (J/g)	*T*_g_ (°C)
A	9.9	114.1	124.8	317.21	97.4
B	12.9	102.6	129.4	628.79	158.3
C	15.9	93.9	121.2	684.36	171.8

**Table 3:** Thermal stability characteristics of the samples obtained from TGA analysis.

Sample Code	Curing Agent (Phr)	*T*_onset_ (°C)	*T*_5%_ (°C)	*T*_max_ (°C)
A	9.9	172.7	257.7	286.6
B	12.9	243.2	309.8	456.2
C	15.9	239.4	297.1	457.9

**Table 4:** Tensile strength, tensile modulus, and elongation at the break of various samples.

Sample Code	Curing Agent (Phr)	Tensile Strength (MPa)	Tensile Modulus (MPa)	Elongation at Break (%)	Izod Impact Strengths (kJ/m^2^)
A	9.9	43.47 ± 2.6	4104.71 ± 38.5	4.46 ± 0.37	27.11
B	12.9	86.91 ± 4.3	3029.64 ± 54.6	3.23 ± 0.62	35.73
C	15.9	79.12 ± 4.7	3467.28 ± 45.8	2.17 ± 0.22	34.29
